# Toward food-grade production of the *Bacteroides helcogenes* protein-glutamine glutaminase with an optimized *Bacillus subtilis* strain

**DOI:** 10.1007/s00253-025-13681-1

**Published:** 2026-01-10

**Authors:** Jana Senger, Mario Keutgen, Nicole Roth, Ines Seitl, Lutz Fischer

**Affiliations:** Institute of Food Science and Biotechnology, Department of Biotechnology and Enzyme Science, University of Hohenheim, Garbenstr 25, 70599 Stuttgart, Germany

**Keywords:** Protein-glutamine glutaminase, *Bacillus subtilis*, CRISPR/Cas9, Production

## Abstract

**Abstract:**

Protein-glutamine glutaminases (PGs; EC 3.5.1.44) have gained attention in the food industry due to their application in plant protein products. The recently discovered PG from *Bacteroides helcogenes* (PGB) has especially been shown to provide promising characteristics for improving the techno-functional properties of plant proteins. A prerequisite for food enzymes, such as the PG, is their production with an expression host that meets food safety and yield requirements. The antibiotic-free and secretory production of the PGB was targeted in this study using the undomesticated *Bacillus subtilis* 007. The CRISPR/Cas9-mediated approach enabled specific genomic PGB integrations, while simultaneously deleting unwanted *B. subtilis* traits. Firstly, the PGB expression cassette was integrated into the *sigF* gene, leading to an asporogenic strain and extracellular activity of 4.1 µkat/L_culture_ in bioreactor cultivations. However, excessive foaming hampered the production process tremendously. Consequently, a second PGB copy was integrated into the *sfp* locus, which is involved in the production of lipopeptides, such as surfactin. As a result, the PGB activity was increased to 5.4 µkat/L_culture_, and foaming during cultivation was reduced significantly. The introduction of a third PGB copy for preventing cell motility did not increase production; however, the integration into the well-established *amyE* locus improved the PGB yield during reactor cultivations. A final extracellular activity of 9.5 µkat/L_culture_ was reached. The multiple genomic integrations of the PGB gene enabled the efficient PGB secretion in an optimized *B. subtilis* host without the need for antibiotics.

**Key points:**

*• Site-specific PGB integration enabled by genome sequencing of B. subtilis 007*.

*• Antibiotic-free and secretory PGB production with an optimized B. subtilis host*.

*• Increased PGB production reaching 9.5 µkat/L*_culture_.

**Supplementary Information:**

The online version contains supplementary material available at 10.1007/s00253-025-13681-1.

## Introduction

The increasing demand for plant-based foods leads to investigations into alternative protein sources. While plant proteins are abundant, certain properties, such as low solubility, limit their application in food production (Day 2013). Enzymatic deamidation by protein-glutamine glutaminases (PGs; EC. 3.5.1.44) is a method used to increase the solubility of proteins (Zhang et al. 2021). The PG catalyzes the conversion of protein-bound L-glutamine into L-glutamic acid, thereby releasing ammonia. Thus, negative charges are introduced, increasing the electrostatic repulsion, which results in the alteration of the protein structure (Chen et al. 2021; Yie et al. 2004). As a result, the protein solubility and other techno-functional properties, such as emulsification or foaming, can be enhanced (Liu et al. 2022; Zhang et al. 2021).

Until now, only a few PGs have been described (Horstmann et al. 2020, 2025; Qu et al. 2022; Yamaguchi et al. 2001). The PG of *Chryseobacterium proteolyticum* (PGC) has especially been intensively studied (Hashizume et al. 2011; Kumeta et al. 2010; Yamaguchi et al. 2001) and is already commercially available (Amano Enzyme Inc., Japan). The PGC is natively secreted as a pre-pro-protein and activated upon pro-sequence cleavage (Hashizume et al. 2011). Horstmann et al. (2020) discovered a new PG of *Bacteroides helcogenes* (PGB). Unlike the PGC, the PGB is active with its pro-sequence, although the activity is remarkably increased by pro-sequence cleavage (Horstmann et al. 2025). Furthermore, the PGB offers several advantages over the PGC, such as higher temperature stability and less product inhibition by ammonia. Therefore, the PGB is especially interesting for application in the food industry (Horstmann et al. 2020).

Enzymes for the food industry, such as the PG, undergo an intensive risk assessment by the European Food Safety Authority, including the production source. Therefore, the production host is intensively characterized to identify traits of concern, such as any antimicrobial resistance genes acquired, virulence factors, or production of toxic metabolites (EFSA CEP Panel 2021). Thus, hosts that have already been pre-evaluated with the Qualified Presumption of Safety status are preferably used for food enzyme production (EFSA Biohaz 2023).

A microbe with Qualified Presumption of Safety status is the Gram-positive bacterium *Bacillus subtilis* (EFSA Biohaz 2023). One key advantage of *B. subtilis* as a host is its capability to secrete enzymes at a gram per liter scale (van Dijl and Hecker 2013), which greatly facilitates further downstream processing. Several enzymes with a potential application in the food industry are produced in a secretory manner with *B. subtilis* (Deckers et al. 2020), such as β-galactosidases, α-amylases, or the PGC (Yao et al. 2019; Yin et al. 2021; Zhao 2018). Interestingly, during PGC secretion, the natively secreted proteases of *B. subtilis* were shown to cleave the pro-sequence of the PGC extracellularly, thereby activating the enzyme (Yin et al. 2021).

In this study, a PGB expression cassette was integrated into the genome of the undomesticated *B. subtilis* 007 using CRISPR/Cas9 for a stable and antibiotic-free PGB production. On the one hand, the high-level PGB expression was targeted through the introduction of multiple gene copies into different loci in the genome. On the other hand, integration sites were selected to simultaneously delete unwanted or energy-consuming features of the host, leading to an optimized *B. subtilis* production strain.

## Material and methods

### Chemicals and enzymes

Chemicals were purchased from Sigma-Aldrich (St. Louis, USA), Carl Roth GmbH (Karlsruhe, Germany), and Fisher Scientific (Hampton, USA). Oligonucleotides for polymerase chain reaction (PCR) were ordered from Biomers (Ulm, Germany). Q5® High-Fidelity DNA Polymerase for the PCR and restriction enzymes for cloning were obtained from New England Biolabs GmbH (Frankfurt am Main, Germany) or Thermo Fisher Scientific (Hampton, USA).

### Strains and media

*Escherichia coli* XL1 Blue (Merck, Darmstadt, Germany) for cloning was cultivated in LB medium (Lennox) with 50 µg/mL kanamycin at 37 °C at 180 rpm. Either 0.5% (w/v) mannose and 5 µg/mL kanamycin or 50 µg/mL kanamycin and 80 µg/mL X-Gal were added for the LB plates. Generated *B. subtilis* strains were grown on LB agar plates at 37 °C overnight. The media described by Zhang et al. (2020) were used for PGB production. Pre-cultures were prepared in seed medium (40 g/L sucrose, 30 g/L soy peptone, 6 g/L KH_2_PO_4_, 2.04 g/L MgCl_2_ × 6 H_2_O). Reactor cultivations were done using fermentation medium (70 g/L sucrose, 50 g/L soy peptone, 5 g/L KH_2_PO_4_, and 3.06 g/L MgCl_2_ × 6 H_2_O). Salts and sucrose were sterilized by filtration (Ø 0.2 µm) and added to the soy peptone, which was autoclaved separately.

### Construction of CRISPR/Cas9 plasmids

The CRISPR/Cas9 plasmids for the genomic integration of the PGB expression cassette were constructed as described previously (Altenbuchner 2016; Watzlawick and Altenbuchner 2019). Plasmids and oligonucleotides used for construction are listed in Supplemental Tab. 1 and 2. Oligonucleotides for the respective sgRNA were designed using CRISPOR (Concordet and Haeussler 2018) with *Bsa*I overhangs. The oligonucleotides were phosphorylated using T4 polynucleotide kinase for 1 h at 37 °C. After phosphorylation, the oligonucleotides were annealed in the following steps: (i) 120 s at 95 °C, (ii) 70 cycles for 45 s at 94–25 °C with a 1 °C decrease in each cycle step, and (iii) cooling at 4 °C. Annealed oligos were ligated into the *Bsa*I-digested pJOE8999 (Altenbuchner 2016) using the T7 ligase and incubation at 20 °C for 1 h. Chemically competent *E. coli* XL1 were transformed with 10 µL of the ligation sample and plated on LB plates with 50 µg/mL kanamycin and X-Gal. After incubation at 37 °C overnight, white colonies were selected, and the plasmids were isolated using the GeneJET Plasmid-Miniprep-Kit (Thermo Fisher Scientific, Hampton, USA). Verification of the correct sgRNA sequence was done by sequencing (Eurofins Genomics, Ebersberg, Germany). Regarding the generation of the repair template, 800–900 bp flanks of the respective target gene (*sigF*, *sfp*, *flgE*, or *amyE*) were amplified by PCR using the Q5 DNA polymerase (Thermo Fisher Scientific, Hampton, USA) and genomic DNA of *B. subtilis* 007 (DSM118688) as a template. The genomic DNA was isolated using the GeneJET genomic DNA Purification Kit (Thermo Fisher Scientific, Hampton, USA). The PGB expression cassette with the native pro-PGB sequence (EMBL-EMI: ADV44662.1; NCBI GenBank Accession: CP002352.1; Protein ID: ADV44662.1) was amplified from plasmid pLF_P_aprE__PhoD_pro-PGB (Supplemental Tab. 1). The comparison of the codon usage is shown in Supplemental Fig. 7. The PCR products with added *Sfi*I sites were purified using the DNA Clean & Concentrator Kit (Zymo Research, Orange, CA) and digested using *Sfi*I at 50 °C for 3–14 h. The pJOE8999 plasmids with integrated sgRNA were digested with *Sfi*I and eventually *Sma*I, and treated with shrimp alkaline phosphatase for 30–60 min at 37 °C. The digested DNA fragments were isolated after 1% (w/v) agarose gel electrophoresis using the GeneJET Gel Extraction-Kit (Thermo Fisher Scientific, Hampton, USA), and ligated at the following ratio: 40 ng expression cassette, 20 ng upstream flank, 20 ng downstream flank, and 100 ng vector. *E. coli* XL 1 were transformed with the ligation sample and plated on LB plates with 50 µg/mL kanamycin. Plasmids were isolated from single colonies and correct integration was verified by digestion and sequencing.

### CRISPR/Cas9 integration of the PGB expression cassette

CRISPR/Cas9 editing was done as described (Altenbuchner 2016; Watzlawick and Altenbuchner 2019), with some modifications. *B. subtilis* strains were transformed with the respective plasmid pJOE8999 after the modified protocol, as described previously (Senger et al. 2025; Vojcic et al. 2012). Transformed cells were plated on LB plates with 0.5% (w/v) mannose and 5 µg/mL kanamycin and incubated overnight at 37 °C or for 2 days at 30 °C. The colonies were streaked again on LB plates with 0.5% (w/v) mannose and 5 µg/mL kanamycin and incubated overnight at 37 °C. Single colonies were streaked on LB plates and incubated for 9–15 h at 50 °C, followed by streaking on LB plates and incubation at 42 °C. Single colonies were screened for plasmid loss due to the temperature-sensitive *ori* by testing the growth on LB with 50 µg/mL kanamycin. Positive clones were tested for the correct integration of the expression cassette by colony PCR using the Taq DNA polymerase Kit (TaKaRa Bio inc., Shiga, Japan). An amount of 50 µL of 20 mM NaOH was inoculated with a single colony, and heated for 10 min at 95 °C. After cooling down the sample, 2 µL of supernatant was used as a template for colony PCR (example in Supplemental Fig. 1). Correct insertion into the *flgE* locus was additionally verified by amplicon sequencing. The PCR product (Supplemental Fig. 1) was purified using the DNA Clean & Concentrator Kit (Zymno Research, Freiburg, Germany) and was sequenced by Eurofins Genomics GmbH (Ebersberg, Germany). The strains constructed (Supplemental Tab. 3) were stored as 30% glycerol stocks at −80 °C.

### Reactor cultivation of *B. subtilis* strains

Reactor cultivations were done to investigate the PGB production of the *B. subtilis* strains constructed using the media described by Zhang et al. (2020). Accordingly, the strains were streaked from glycerol stocks on LB plates. Single colonies were used for the inoculation of 10 mL of seed medium in 100-mL flasks and incubated for 9–15 h at 37 °C at 180 rpm. The first preculture was used for the inoculation of the second preculture (190 mL seed medium in 1-L flasks) and incubation was done at 37 °C at 100 rpm for 14 h. The reactor cultivations were done using the 1-L Multifors bioreactor systems (Infors, HT) equipped with 405-DPAS-SC-K8S pH and InPro 6900 sensors of Mettler Toledo for the detection of pH and pO_2_, respectively. The temperature and aeration rate were kept at 30 °C and 1 vvm. The pH was regulated by the addition of 2 M NaOH and 2 M H_3_PO_4_, and pO_2_ was held above 30% by increasing the stirrer speed stepwise. A working volume of 800 mL was used for the batch cultivation. Cultivations were done at least in biological duplicate. An amount of 80 mL of preculture was used to inoculate 720 mL of medium in the bioreactor. Cultivation was done for 56 h. If required, Antifoam 204 (Sigma Aldrich, USA) was added as an antifoaming reagent. A “foam trap” was applied to prevent culture loss for the cultivation of *B. subtilis* FS1 (Supplemental Fig. 2). Samples were taken periodically and OD_600_, bio dry mass (BDM), extracellular peptidase, and PG activity were determined. All measurements were done at least in triplicate.

### Determination of PG activity

Cultivation samples were centrifuged for 5 min at 13,000 g and 4 °C. The supernatant was applied on PD MidiTrap G-25 columns (Cytiva, MA, USA) and eluted in activity buffer (100 mM 2-(*N*-Morpholino) ethane sulfonic acid hydrate, pH 5.5) for PG activity determination using the synthetic substrate Z-Gln-Gly-OH (Horstmann et al. 2025). Accordingly, 122 µL of activity buffer was mixed with 18 µL of 200 mM Z-Gln-Gly-OH and incubated for 5 min at 50 °C and 800 rpm. Similarly, the PG sample solution was pre-incubated at 50 °C and 800 rpm for 5 min. The reaction was initiated by adding 40 µL of the PG solution to the buffer-substrate solution, after which the mixture was incubated for 10–60 min at 50 °C and 800 rpm. The reaction was stopped by the addition of 20 µL 100 mM mercury chloride and the samples were centrifuged for 5 min at 13,000 g and 4 °C. The formation of Z-Glu-Gly-OH from Z-Gln-Gly-OH was determined by reverse-phase high-pressure liquid chromatography equipped with a C18 column (Eurosil Bioselect 300-5 (125 × 4.6 mm), Knauer, Germany), DAD detector, and measured at 205 nm. Separation was done using a gradient of eluent A (0.1% (v/v) trifluoracetic acid in H_2_O_dd_) and eluent B (0.1% (v/v) trifluoroacetic acid in acetonitrile) with a constant flow of 1 mL/min. The concentrations of eluent B increased gradually: 0–2 min 10–25% eluent B, 9–10 min 25% B, 10–11 min 25–60% B, 11–14 min 60% B, 14–15 min 60–10% B, and 15 18 min 10% B. An amount of 0.5 mM hippuric acid was used as the internal standard. One katal of PGB activity was defined as the production of 1 mol of Z-Glu-Gly-OH per second.

### Determination of peptidase activity

The peptidase activity in the culture supernatant was determined using 2.5 g/L azocasein (Megazyme International, Wicklow, Ireland) as the substrate. Cultivation samples were centrifuged for 5 min at 13,000 g and 4 °C. The supernatant was applied on PD MidiTrap G-25 columns (Cytiva, MA, USA) and eluted in 25 mM NaPO_4_ with pH 7.0. An amount of 250 µL of azocasein and desalted sample were incubated separately at 30 °C at 800 rpm for pre-incubation. After 5 min, the reaction was started by the addition of 50 µL sample to the azocasein solution and the incubation was done at 30 °C for 10 min at 800 rpm. The reaction was stopped by the addition of 25 µL 2 M trichloroacetic acid. After centrifugation at 13,000 g for 5 min and 4 °C, 190 µL sample was transferred to a microtiter plate and 60 µL of 1 M NaOH was added. The absorption was measured at 450 nm and the peptidase activity was calculated as the absorption difference per hour and mL.

### Protein content determination and SDS PAGE

The protein content in the supernatant during cultivation was determined according to Bradford (1976) using bovine serum albumin as the standard. The secretome of the *B. subtilis* strains was analyzed by 12.5% sodium dodecyl sulfate polyacrylamide gel electrophoresis (Laemmli 1970) with the addition of 6 µL of buffer-exchanged supernatant mixed with 5 × loading buffer (0.02% (w/v) Tris-HCl, 6% (w/v) glycerol, 0.1% (w/v) bromophenol blue, 4% (w/v) SDS, and 2% (w/v) β-mercaptoethanol). The Color Precision Plus Protein™ Unstained protein standard (1610363, Bio-Rad Laboratories Inc., CA, USA) was used as a reference. The visualization of proteins was done by Coomassie Brilliant Blue G-250 staining (Fairbanks et al. 1971).

### Genome sequencing and in silico analyses

The *B. subtilis* genomic DNA was isolated using the GeneJET genomic DNA Purification Kit (Thermo Fisher Scientific, Hampton, USA). Afterwards, the DNA was concentrated using the Genomic DNA Clean & Concentrator Kit (Zymno Research, Freiburg, Germany). The genome sequencing and annotation were conducted by Eurofins Genomics GmbH (Ebersberg, Germany) using Illumina sequencing and the Prokka software v1.13.3 (Seemann 2014). The analyses and visualization of the *B. subtilis* genome were done utilizing Artemis (Carver et al. 2012). JSpeciesWS (Richter et al. 2016) was used for pairwise comparisons of genomes, and protein sequence alignments were done using ClustalO (Sievers and Higgins 2014).

## Results

### Genome sequence of the undomesticated strain *B. subtilis* 007

*B. subtilis* 007 is an undomesticated strain isolated from compost and has already been found suitable for recombinant enzyme production (Senger et al. 2025). Moreover, this strain showed enhanced PGB secretion and activation compared to commonly used domesticated *B. subtilis* strains in preliminary experiments (not shown). To enable genomic engineering and establish *B. subtilis* 007 as a production host, the genome was sequenced. After *de novo* assembly, 113 contigs were obtained, leading to a genome size of 4,193,295 bp with a GC content of 43.37% (NCBI Accession number JBNKQS000000000; Genome map Supplemental Fig. 3). A pairwise comparison of the *B. subtilis* 007 genome to the widely used laboratory strain *B. subtilis* 168 and several other *B. subtilis* strains (Table 1) revealed the highest identity to *B. subtilis* NRS6181 and MB8_B10 (highlighted in bold).

**Table 1:** Comparison of the *B. subtilis* 007 genome to other *B. subtilis* strains. The average nucleotide identity determined through BLAST+ (ANIb) and MUMmer (ANIm) is displayed for pairwise comparison

Strain	Size [bp]	GC [%]	ANIb	ANIm	GenBank no.
168	4,215.606	43.5	98.56	98.79	AL009126.3
ATCC 6051	4,215,610	43.5	98.56	98.79	CP003329
NCBI 3610	4,215,607	43.5	98.56	98.79	CP020102
PY79	4,033,459	43.8	98.51	98.79	CP006881
W23	4,027,676	43.9	92.17	92.84	CP002183
SMY	4,214,643	43.5	98.53	98.76	CM000490
BSn5	4,093,599	43.8	98.50	98.90	CP002468
BSP1	4,043,754	43.9	98.53	98.88	CP003695
KCTC 1028	4,215,633	43.5	98.57	98.79	CP011115
HMNig-2	4,178,124	43.6	98.16	98.57	CP031784
**MB8_B10**	**4,225,362**	**43.5**	**99.42**	**99.59**	**CP045824**
MB9_B1	4,263,919	43.5	99.07	99.20	CP045820
MENO2	4,083,694	43.8	98.30	98.52	CP031783
P5_B2	4,103,324	43.6	98.16	98.68	CP04581
TO-A JPC	4,090,708	43.8	98.62	98.85	CP011882
SP1	4,027,676	43.5	98.56	98.79	CP058242
3NA	4,195,102	43.6	98.55	98.78	CP010314
JH642 AG174	4,188,369	43.5	98:49	98.79	CP007800
**NRS6181**	**4,189,561**	**43.7**	**99.77**	**99.99**	**OX419555**
UD1022	4,027,676	43.9	97.98	98.32	CP011534

Similar to *B. subtilis* 007, *B. subtilis* NRS6181 and MB8_B10 are undomesticated strains that were isolated from soil in the UK and Denmark, respectively (Kalamara et al. 2021; Kiesewalter et al. 2020). Interestingly, *B. subtilis* MB8_B10 has been shown to produce the non-ribosomally synthesized lipopeptides surfactin and plipastatin (Kiesewalter et al. 2021), which can cause strong foaming during cultivation (Pardhi et al. 2022). These lipopeptides are synthesized by non-ribosomal peptide synthetases that require activation by the 4-phosphopantetheinyl transferase Sfp (Quadri et al. 1998). Unlike *B. subtilis* MB8_B10, the domesticated strain *B. subtilis* 168 has lost the ability to produce surfactin due to an inactive *sfp* gene (Nakano et al. 1988, 1992). However, *B. subtilis* 007 contains a functional *sfp* gene (Supplemental Fig. 8) and the gene clusters for surfactin and plipastatin synthetases, indicating the strain’s ability to produce these lipopeptides. Surfactin and plipastatin probably cause the excessive foaming observed during the cultivation of *B. subtilis* 007 (Supplemental Fig. 2B).

### Genomic PGB integrations using CRISPR/Cas9 for the optimization of *B. subtilis* 007

**Fig. 1:**
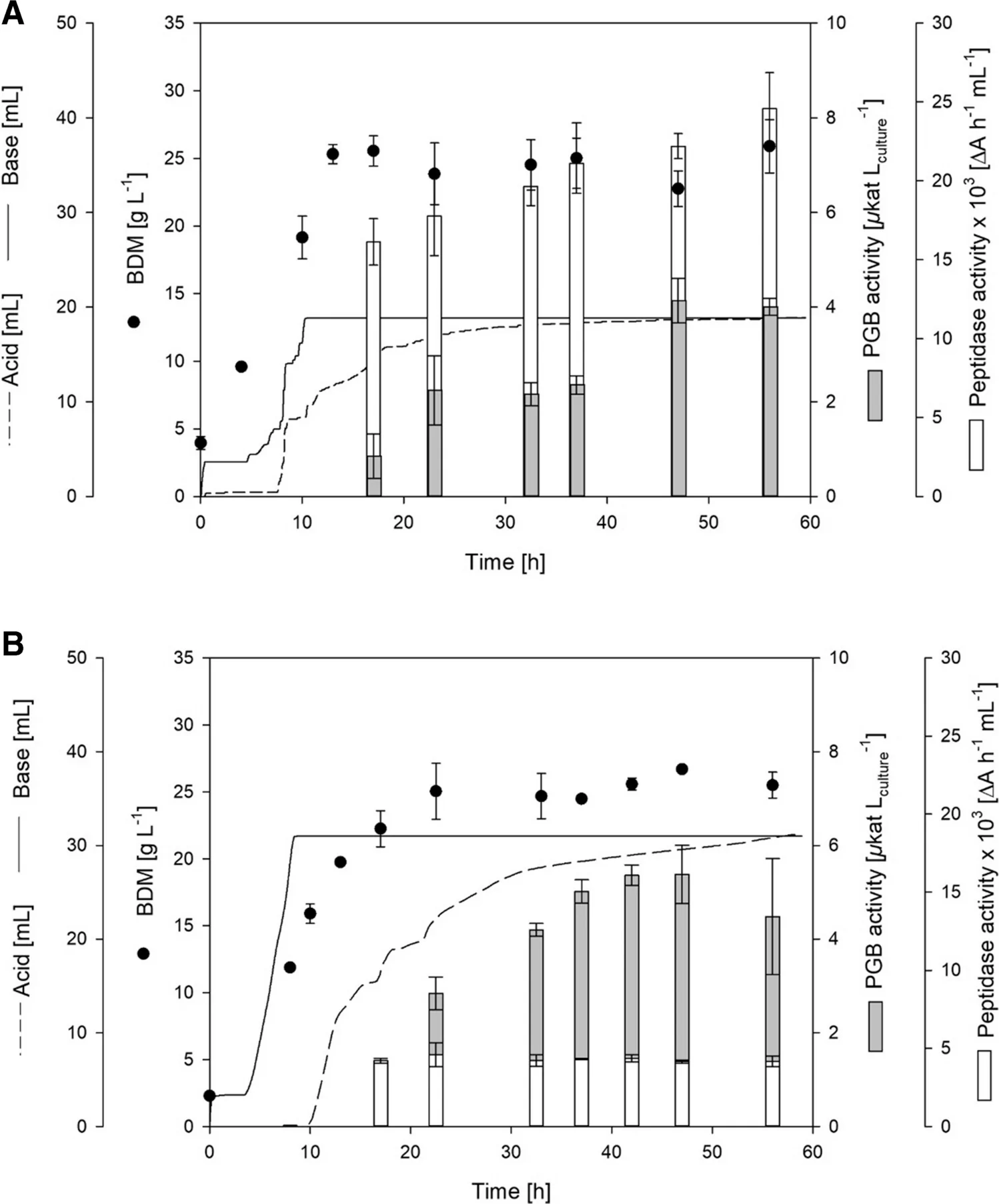
Bioreactor cultivation of *B. subtilis* FS1 (**A**) and FS2 (**B**) for secretory PGB production. BDM, PGB activity, and peptidase activity were determined after 8, 17, 22, 32, 37, 42, 47, and 56 h of cultivation

The expression cassette suitable for PGB secretion consisted of the P_aprE_ promoter, the native pro-PGB gene fused to the PhoD signal peptide of *B. subtilis* (Kolkman et al. 2008), and the BPN’ terminator of *Bacillus amyloliquefaciens*. The genome sequence of *B. subtilis* 007 enabled the specific integration of the PGB expression cassette into the genome. Integration sites were selected to simultaneously optimize the *B. subtilis* production strain by deleting unwanted genes and features. *SigF* was selected as the first target gene. SigF is the sigma factor for the early stages of sporulation, controlling the expression of forespore genes (Riley et al. 2020). Consequently, deletion of *sigF* in *B. subtilis* 007 due to the integration of the PGB expression cassette led to an asporogenic phenotype of the constructed strain *B. subtilis* FS1 (Supplemental Fig. 5 A). Subsequently, *B. subtilis* FS1 was cultivated in a batch reactor cultivation (Fig. 1A; Table 2).

**Table 2:** Comparison of the cultivation of the engineered *B. subtilis* strains FS1–FS4. For each strain, the cultivation time point at which the highest PGB activity was observed (either at 37 or 47 h) was selected for the comparison of bio dry mass (BDM) and activity

Strain	PGB copy	Time [h]^a)^	Vol. PGB activity [µkat/L]	BDM [g/L]	Spec. PGB activity [µkat/g_BDM_]	Peptidase activity × 10^3^ [ΔA h/mL]
FS1	1	47	4.1 ± 0.5	22.8 ± 1.3	0.18	22.2 ± 0.8
FS2	2	47	5.4 ± 0.6	26.7 ± 0.1	0.20	4.2 ± 0.1
FS3	3	37	2.5 ± 0.3	25.3 ± 0.9	0.10	1.8 ± 0.1
FS4	3	47	9.5 ± 0.2	29.5 ± 0.1	0.32	6.9 ± 0.1

^a)^Cultivation time of the highest PGB activity

The cells reached the stationary phase after 13 h with a BDM of 25.3 g/L. The PGB activity increased strongly when the cells entered the stationary phase, which corresponds to the enhanced promoter activity during this growth phase (Jan et al. 2000). The highest extracellular PGB activity was reached after 47 h with 4.1 ± 0.1 µkat/L_culture_ resulting in a specific PGB activity of 0.18 µkat/g_BDM_ (Table 2). In addition, the determination of the peptidase activity should allow conclusions about the activity of the P_aprE_ promoter applied as AprE is one of the main extracellular *B. subtilis* proteases (Harwood and Kikuchi 2022). The endopeptidase activity increased in the stationary phase from 16.1 × 10^3^ up to 24.6 × 10^3^ ΔA h/mL at the end of cultivation (Fig. 1A).

Excessive foaming was observed during cultivation; this hampered the production and sampling, and could not be reduced by the addition of antifoam. Consequently, the lost culture was continuously pumped back into the reactors (Supplemental Fig. 2) to prevent the loss of the cultivation broth and secreted PGB. However, this setup was not feasible for high-level production. It was hypothesized that excessive foaming using *B. subtilis* 007 is caused by the functional *sfp* gene leading to the production of non-ribosomally synthesized lipopeptides. Consequently, *sfp* was deleted in *B. subtilis* FS1 by the integration of the PGB expression cassette, leading to *B. subtilis* FS2. The cultivation of *B. subtilis* FS2 in the bioreactors resulted in a similar growth to *B. subtilis* FS1, with the highest BDM of 26.7 ± 0.1 after 47 h (Fig. 1B, Table 2). Foaming was remarkably reduced, enabling easier process handling (Supplemental Fig. 5B). In addition, extracellular PGB activity was improved to 5.4 ± 0.6 µkat/L after 47 h. Surprisingly, extracellular endopeptidase activity was strongly reduced to 4.2 × 10^3^ ΔA h/mL, which was fivefold lower than in *B. subtilis* FS1, indicating broad regulatory changes upon *sfp* deletion.

### Flagella disruption reduced secretory PGB production

*B. subtilis* can adapt to environmental changes by differentiating into motile cells facilitated by rotating flagella (Lopez et al. 2009; Wadhwa and Berg 2022). The flagella are set up by a filament connected to the motor by the “hook” structure encoded by *flgE* (Mukherjee and Kearns 2016). Fehler et al. (2022) disrupted the motility by deleting *flgE*, which is part of the *fla*/*che* operon. Thus, secretory amylase production increased by about 30% as the energy-consuming cell process of motility was eliminated. In order to investigate whether flagella disruption improves the secretory PGB production, the PGB expression cassette was integrated into the *fla*/*che* operon, thereby deleting *flgE*. Correct integration was verified by colony PCR and amplicon sequencing (Supplemental Fig. 1). The *B. subtilis* FS3 strain generated was cultivated in bioreactors. Growth was comparable to the other strains (Table 2; Supplemental Fig. 4), but extracellular PGB activity was not increased. A maximum activity of only 2.5 ± 0.3 µkat/L_culture_ was reached after 37 h, which was about twofold lower than production in *B. subtilis* FS2. In contrast to the literature, the *flgE* deletion did not improve the activity in this study. Similarly, the extracellular peptidase activity was slightly decreased, yielding only 2.3 × 10^3^ ΔA h/mL at the end of cultivation (Supplemental Fig. 4). Consequently, *B. subtilis* FS3 was not pursued any further.

### Increasing the gene copy number for enhanced PGB production

**Fig. 2:**
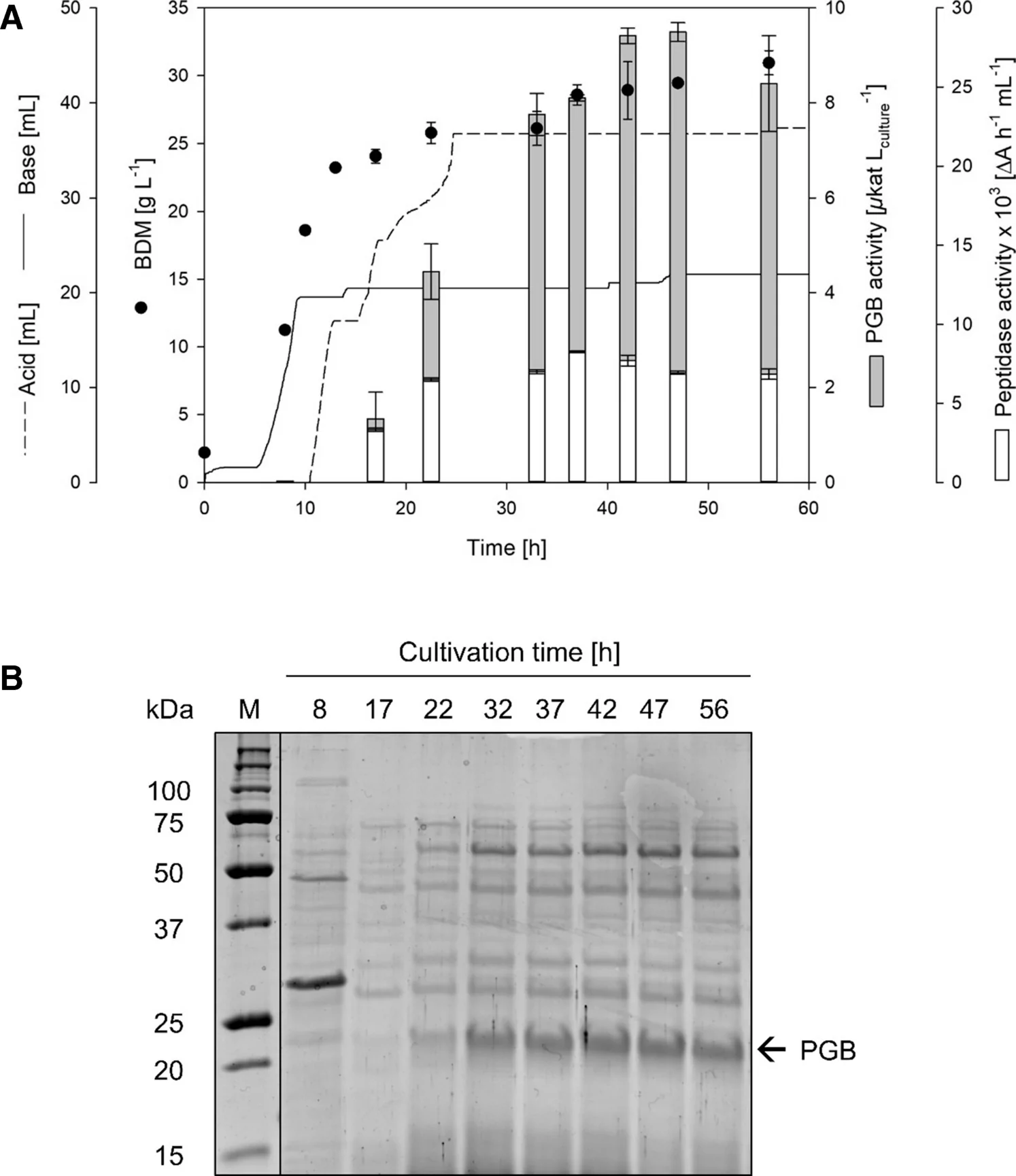
Bioreactor cultivation (**A**) of *B. subtilis* FS4 and SDS PAGE (**B**) of supernatant samples taken at different time points [h]. Sampling was done after 8, 17, 22, 32, 37, 42, 47, and 56 h of cultivation

Two PGB gene copies had already been integrated into the genome in *B. subtilis* FS2. In order to further enhance the PGB production, a third expression cassette was integrated into *B. subtilis* FS2 since enzyme production yield depends strongly on the expression level, which can be influenced by increasing the gene copy number. The PGB expression cassette was integrated into the *amyE* locus, yielding *B. subtilis* FS4. The *amyE* gene encodes the nonessential α-amylase and is an established integration site for stable expression (Härtl et al. 2001; Hidenori and Henner 1986; Jeong et al. 2018). *B. subtilis* FS4 was cultivated in bioreactors and reached the highest BDM of 30.9 ± 1 after 56 h (Table 2; Fig. 2A). Extracellular peptidase activity had already been detected after 17 h and was stable around 6.9 × 10^3^ ΔA h/mL during the stationary phase. The extracellular PGB activity was remarkably improved, yielding 9.5 ± 0.2 µkat/L_culture_ after 47 h (Fig. 2A, Table 2). This result was supported by SDS PAGE (Fig. 2B), where a band of approximately 21 kDa was observed. This dominant band corresponded to the mature PGB, which was verified previously (Supplemental Fig. 6).

## Discussion

In this study, the undomesticated *B. subtilis* 007 was optimized for recombinant PGB production. The PGB has a potential application in food processing (Horstmann et al. 2020), thus, an antibiotic-free PGB production is desired to meet the requirements for food enzymes. Accordingly, the PGB expression cassette was integrated into the genome of *B. subtilis* 007 using CRISPR/Cas9, constructing an expression system without any acquired antibiotic resistance genes.

Sequencing the genome of *B. subtilis* 007 enabled genetic engineering and revealed a high genetic similarity to other undomesticated *B. subtilis* isolates (Table 1). Undomesticated *B. subtilis* exhibits ancestral traits, such as biofilm formation, surfactin production, or swarming motility, which have been lost in laboratory strains, such as *B. subtilis* 168, through domestication (Barreto et al. 2020; McLoon et al. 2011; Zeigler et al. 2008). *B. subtilis* 168 acquired various mutations throughout the laboratory cultivation; these resulted in the loss of gene functionalities, such as *sfp*, *swrA*, or *gudB* (McLoon et al. 2011; Zeigler et al. 2008). Some of these ancestral traits in the undomesticated *B. subtilis* 007 might be beneficial for recombinant enzyme production. A functional *gudB* gene (Supplemental Fig. 8; Belitsky and Sonenshein 1998), for instance, encoding a glutamate dehydrogenase improved the growth of the undomesticated *B. subtilis* ATCC6051 in complex medium (Kabisch et al. 2013). Other traits, such as surfactin production through a functional *sfp* gene (Supplemental Fig. 8), can hamper the production process.

Unfavorable *B. subtilis* 007 genes were targeted as integration sites for the PGB expression cassette, thereby eliminating the respective characteristic. Construction of an asporogenic strain was desired since sporulation represents a contamination risk and additionally limits the recombinant enzyme yields by wasting energy and nutrients (Bressuire-Isoard et al. 2018). Therefore, the sigma factor for the early stages of sporulation (SigF) was deleted. Disruption of *sigF* has already been shown to be feasible in an isolated *Bacillus* strain (Zhang et al. 2016). Additionally, no negative effects on P_aprE_ activity were reported for the *sigF* deletion, in contrast to disruptions of other sporulation genes, such as *spo0A* (Ferrari et al. 1988; Olmos et al. 1996; Overkamp and Kuipers 2015). The integration of the PGB expression cassette into *sigF* (*B. subtilis* FS1) led to a non-sporulating strain and enabled the successful PGB secretion with only a single gene copy.

In contrast to *B. subtilis* 168 derivatives, *B. subtilis* 007 possesses a functional *sfp* gene encoding the 4-phosphopantetheinyl transferase Sfp (Quadri et al. 1998). Sfp is essential for the synthesis of non-ribosomally synthesized peptides, such as surfactin (Nakano et al. 1992). Surfactin is an amphiphilic lipopeptide leading to severe foam formation during cultivation. The production of surfactin is additionally stimulated through foaming and growth on sucrose (Alonso and Martin 2016; Tian et al. 2021). Consequently, the integration of a second PGB copy into the *sfp* locus greatly reduced foaming during cultivation, resulting in an improved fermentation process. Interestingly, the extracellular peptidase activity was fivefold lower than in *B. subtilis* FS1. Surfactin can act as a signaling molecule triggering Spo0A phosphorylation (López et al. 2008; Rahman et al. 2021). Spo0A is a master regulator involved in various cell differentiation processes (Molle et al. 2003) and phosphorylated Spo0A activates the production of the major extracellular proteases by repression of the respective transcriptional repressor AbrB (Strauch and Hoch 1993). It has been speculated that the inhibited surfactin production might lead to a reduced Spo0A phosphorylation and, thus, reduced extracellular protease activity by lowering the AbrB repression. However, the PGB expression in this study was controlled by the P_aprE_ promoter, which is the native promoter of one of the major extracellular proteases (Jan et al. 2000). Consequently, the promoter would underlie the same regulatory changes as the extracellular protease production, leading to reduced P_aprE_ activity. Surprisingly, the PGB production in *B. subtilis* FS2 was increased, indicating a higher production due to the second PGB copy introduced into the genome. As the expression cassette was integrated downstream of the Sfp promoter region, a dual promoter effect is likely to strengthen the PGB production, as has been speculated in other studies (Jeong et al. 2018).

The motility of cells by flagella is used to exploit any available nutrients. However, this energy-expensive cell process is considered dispensable in the laboratory environment and represents a waste of cellular resources. Furthermore, the increased secretory enzyme production observed upon flagella deletion (Fehler et al. 2022) suggested the *flgE* locus as a target site for improving PGB production. The *flgE* gene is part of the *fla*/*che* operon, whose expression is controlled by numerous regulators (Mukherjee and Kearns 2016). In contrast to the literature, replacing *flgE* with the PGB expression cassette did not improve production in this study. Fehler et al. (2022) used a domesticated *B. subtilis* strain for their experiments, which lacked both a functional *sfp* gene and SwrA, the master activator of flagellar biosynthesis. Sfp and predominantly SwrA activate the expression of the *fla*/*che* operon (Ghelardi et al. 2012; Mukherjee and Kearns 2016). In contrast to the strain used by Fehler et al. (2022), *B. subtilis* 007 contains a functional *sfp* and *swrA* gene (Supplemental Fig. 8). It was assumed that different regulations of the *fla/che* operon between *B. subtilis* 007 and the strain used by Fehler et al. resulted in differences in secretory enzyme production. Moreover, Fehler et al. (2022) showed that CRISPRi-mediated knockdown of the *fla*/*che* operon improved amylase production tenfold more than *flgE* deletion. Thus, they suggested unknown, extensive regulatory changes within the cell. Conclusively, the reduced PGB production upon *flgE* deletion in this study is probably due to the different genetic background of *B. subtilis* 007 along with unknown regulatory changes. Further research is required to investigate these intriguing physiological differences.

In order to increase the gene copy number, the PGB expression cassette was integrated into the *amyE* locus. Integration of a third gene copy into the well-established *amyE* locus enhanced the extracellular PGB activity to 9.5 µkat/L. The *amyE* locus is located close to the *ori*, which can further increase the gene copy number, as has been described for fast-growing *B. subtilis* cells (Jeong et al. 2018; Sauer et al. 2016). As the PGB expression cassettes were integrated downstream of the respective promoters, again, a dual promoter effect can contribute to the high PGB production in *B. subtilis* FS4.

The strategy of multiple PGB integrations into the genome of *B. subtilis* 007 led to an efficient PGB secretion and activation (Fig. 2). The highest PGB activity of 9.5 µkat/L in *B. subtilis* FS4 was almost 300-fold higher than previous intracellular pro-PGB production in *E. coli* BL21 with 34 nkat/L_culture_ and did not require an additional activation step for pro-sequence cleavage (Horstmann et al. 2025). Furthermore, disadvantages of plasmid-based expression systems, such as plasmid stability (Fleming and Patching 1994; Shoham and Demain 1991), the use of antibiotic resistance markers, or heterogeneity due to polar fixation and copy number variations (Mileyko et al. 2008; Münch et al. 2015), were circumvented. Furthermore, the PGB integration enabled the deletion of unwanted features of *B. subtilis* 007, resulting in the construction of an optimized production host. Based on *B. subtilis* FS4, further strain optimizations are possible. Mutating transcriptional regulators in *B. subtilis*, such as DegU, for instance, can increase the P_aprE_-controlled expression and contribute to the homogeneity in the population (Dahls et al. 1992; Ploss et al. 2016). Moreover, the deletion of autolysins can improve cell growth (Kabisch et al. 2013), leading to higher cell densities and probably higher enzyme amounts. A common strategy to reduce extracellular proteolysis is the deletion of extracellular proteases (Harwood and Kikuchi 2022). However, some of these proteases are essential for pro-sequence cleavage of the PGB and thus must be identified first. This was clearly demonstrated in the study of Ouyang et al. (2021), who produced the PGC in different *B. subtilis* strains: The highest extracellular PGC activity of 0.6 U/mL (= 10 µkat/L) was reached with *B. subtilis* 168. The three-protease knock-out strain *B. subtilis* DB403 also enabled secretion of active PGC, yielding 0.5 U/mL (= 8.3 µkat/L). In contrast, extracellular PGC activation was not possible in *B. subtilis* WB800N, which lacks eight extracellular proteases (Ouyang et al. 2021).

In addition to the strain engineering, optimization of the cultivation conditions by establishing a fed-batch process instead of batch cultivation can be a powerful strategy to increase the secretory PGB production with *B. subtilis* 007.

## Conclusion

The stable and antibiotic-free PGB production via genomic integration using the CRISPR/Cas9-mediated approach in the undomesticated *B. subtilis* 007 was realized for the first time. The straightforward approach of successively integrating multiple copies of the PGB expression cassette into the genome increased the secretory PGB production and optimized the *B. subtilis* host simultaneously. Thereby, the choice of the genomic integration site had a remarkable effect on the production. This study paves the way for further improvements in the production process aiming at food-grade PGB production.

## Supplementary Information

Below is the link to the electronic supplementary material.

ESM 1 (PDF 975 KB)

## Data Availability

All data generated or analyzed during this study are included in this published article [and its supplementary information files].
