# Supplementary material for: Toward food-grade production of the Bacteroides helcogenes protein-glutamine glutaminase with an optimized Bacillus subtilis strain
Source: Appl Microbiol Biotechnol. 2026 Jan 10;110(1):8. doi: 10.1007/s00253-025-13681-1 (PMC12791059; doi:10.1007/s00253-025-13681-1)
Supplement: Supplementary file 1 — (PDF 975 KB) [file 253_2025_13681_MOESM1_ESM.pdf]

Supplemental material

Toward food-grade production of the *Bacteroides helcogenes* protein-glutamine glutaminase with an optimized *Bacillus subtilis* strain

Jana Senger, Mario Keutgen, Nicole Roth, Ines Seitl, Lutz Fischer*

University of Hohenheim, Institute of Food Science and Biotechnology,

Department of Biotechnology and Enzyme Science, Garbenstr. 25, 70599 Stuttgart, Germany

*Corresponding author

E-mail address: lutz.fischer@uni-hohenheim.de

Tel.: +49 711 459 22311

Supplemental Table 1: Plasmids used in this study.

Plasmid	Description
pLF_P_{aprE}_ PhoD_pro-PGB	Plasmid with PGB expression cassette (=pA-PGB) with expression controlled by P_{aprE} and pro-PGB gene (EMBL-EMI: ADV44662.1) fused to the PhoD signal peptide.
pJOE8999	CRISPR/Cas9 plasmid for *B. subtilis* (Altenbuchner 2016)
pJOE8999_sgSigF	CRISPR/Cas9 plasmid with integrated sgRNA for *sigF*
pJOE8999_sgSigF_pA-PGB	CRISPR/Cas9 plasmid with integrated sgRNA for *sigF* and PGB expression cassette
pJOE8999_sgSfp	CRISPR/Cas9 plasmid with integrated sgRNA for *sfp*
pJOE8999_sgSfp_pA-PGB	CRISPR/Cas9 plasmid with integrated sgRNA for *sfp* and PGB expression cassette
pJOE8999_sgFlgE	CRISPR/Cas9 plasmid with integrated sgRNA for *flgE*
pJOE8999_sgFlgE_pA-PGB	CRISPR/Cas9 plasmid with integrated sgRNA for *flgE* and PGB expression cassette
pJOE8999_sgAmyE	CRISPR/Cas9 plasmid with integrated sgRNA for *amyE*
pJOE8999_sgAmyE_pA-PGB	CRISPR/Cas9 plasmid with integrated sgRNA for *amyE* and PGB expression cassette

Supplemental Table 2: Oligonucleotides used in this study.

	Oligonucleotide	Sequence 5'→3'	Application
1	SigF_O1	TACGACAGCCCGATGCAG CCGATC	sgRNA for *sigF* locus, *Bsa*I overhang
2	SigF_O2	AAACGATCGGCTGCATCG GGCTGT	sgRNA for *sigF* locus, *Bsa*I overhang
3	Sfp_O1	TACGGCACATCTCCCAGC AGGGTG	sgRNA for *sfp* locus, *Bsa*I overhang
4	Sfp_O2	AAACCACCCTGCTGGGAG ATGTGC	sgRNA for *sfp* locus, *Bsa*I overhang
5	Flg_O1	TACGCACAAGACGGGGGA CAAATC	sgRNA for *flgE* locus, *Bsa*I overhang
6	Flg_O2	AAACGATTTGTCCCCCGTC TTGTG	sgRNA for *flgE* locus, *Bsa*I overhang
7	AmyE_O1	TACGTGAAGATCAGGCTAT CACTG	sgRNA for *amyE* locus, *Bsa*I overhang
8	AmyE_O2	AAACCAGTGATAGCCTGAT CTTCA	sgRNA for *amyE* locus, *Bsa*I overhang
9	RepairPG_Fw_SfI	AAGGCCATCGTGGCCGAA	Amplification of PGB expression cassette
10	RepairPG_Rev_SfI	AAGGCCTTCAGGGCCCGA TTACGAATGCCGTCTC	Amplification of PGB expression cassette
11	Up_flank_sigF_for	AAGGCCAACGAGGCCGCA TGAGCCTTGGAATTGAC	Amplification *sigF* upstream flank
12	Up_flank_sigF_rev	AAGGCCACGATGGCCTCT CCTTAATTACAAAGCGC	Amplification *sigF* upstream flank
13	Down_flank_sigF_for	AAGGCCCTGAAGGCCCTA GTCTGCAGTGCAGGCTAG	Amplification *sigF* downstream flank

Continuation Supplemental Table 2			
	**Oligonucleotide**	**Sequence 5'→3'**	**Application**
14	Down_flank_sigF_rev	AAGGCCTTATTGGCCCAC CGTTATGCCTCCGCCG	Amplification *sigF* downstream flank
15	Up_flank_sfp_for	AAGGCCAACGAGGCCCTG TCAGATGTGCTACAATGAC	Amplification *sfp* upstream flank
16	Up_flank_sfp_rev	AAGGCCACGATGGCCCAT TCTAGATCCTCCGTCTGC	Amplification *sfp* upstream flank
17	Down_flank_sfp_for	AAGGCCCTGAAGGCCGCT CATCAACAGCTTGACAC	Amplification *sfp* downstream flank
18	Down_flank_sfp_rev	AAGGCCTTATTGGCCGAA GATCGCCATTGAACAGC	Amplification *sfp* downstream flank
19	Up_flank_Flg_for	AAGGCCAACGAGGCCGCA GATGTCATCTGATATAC	Amplification *flgE* upstream flank
20	Up_flank_Flg_rev	AAGGCCACGATGGCCCCA GAATAAAGTGAACGTAAC	Amplification *flgE* upstream flank
21	Down_flank_Flg_for	AAGGCCCTGAAGGCCCAT CTGATGAAATCCTTCAAG	Amplification *flgE* downstream flank
22	Down_flank_Flg_rev	AAGGCCTTATTGGCCGAG TGGTAAGAAGTCTTGC	Amplification *flgE* downstream flank
23	Up_flank_AmyE_for	AAGGCCAACGAGGCCGGG CTTGTCTTTATCGTG	Amplification *amyE* upstream flank
24	Up_flank_AmyE_rev	AAGGCCACGATGGCCCCT GATGTGAAGACTGGA	Amplification *amyE* upstream flank
25	Down_flank_AmyE_for	AAGGCCCTGAAGGCC CAATGTGATGGCTGGACA G	Amplification *amyE* downstream flank
26	Down_flank_AmyE_rev	AAGGCCTTATTGGCC CTCAATGAGGAAGAGAAC CG	Amplification *amyE* downstream flank
27	CC_AmyE	CTGTCCTTGCCGGTTTAAT AG	Colony PCR
28	PG-seq 16/60	GCTGAAGATACATCCAG	Colony PCR
29	CC_FlgE	CTGACAGATATGTGAATAT AGG	Colony PCR
30	CC_sigF	GCCTGAACAAATCTCATTG	Colony PCR
31	CC_sfp	GAACGGCATATTTTCATTA C	Colony PCR
32	Seq_sgRNA_pJOE8999	GACCTCAAAAAGGTCTTTA	Sequencing sgRNA in pJOE8999

Supplemental Table 3: *B. subtilis* strains used in this study.

Strain designation	Genotype	Reference
B. subtilis 007	Wild-type isolate	DSM118688
B. subtilis FS1	*B. subtilis* 007 Δ*sigF*::pA_PGB	This study
B. subtilis FS2	*B. subtilis* 007 Δ*sigF*::pA_PGB Δ*sfp*::pA_PGB	This study
B. subtilis FS3	*B. subtilis* 007 Δ*sigF*::pA_PGB Δ*sfp*::pA_PGB Δ*flgE*::pA_PGB	This study
B. subtilis FS4	*B. subtilis* 007 Δ*sigF*::pA_PGB Δ*sfp*::pA_PGB Δ*amyE*::pA_PGB	This study

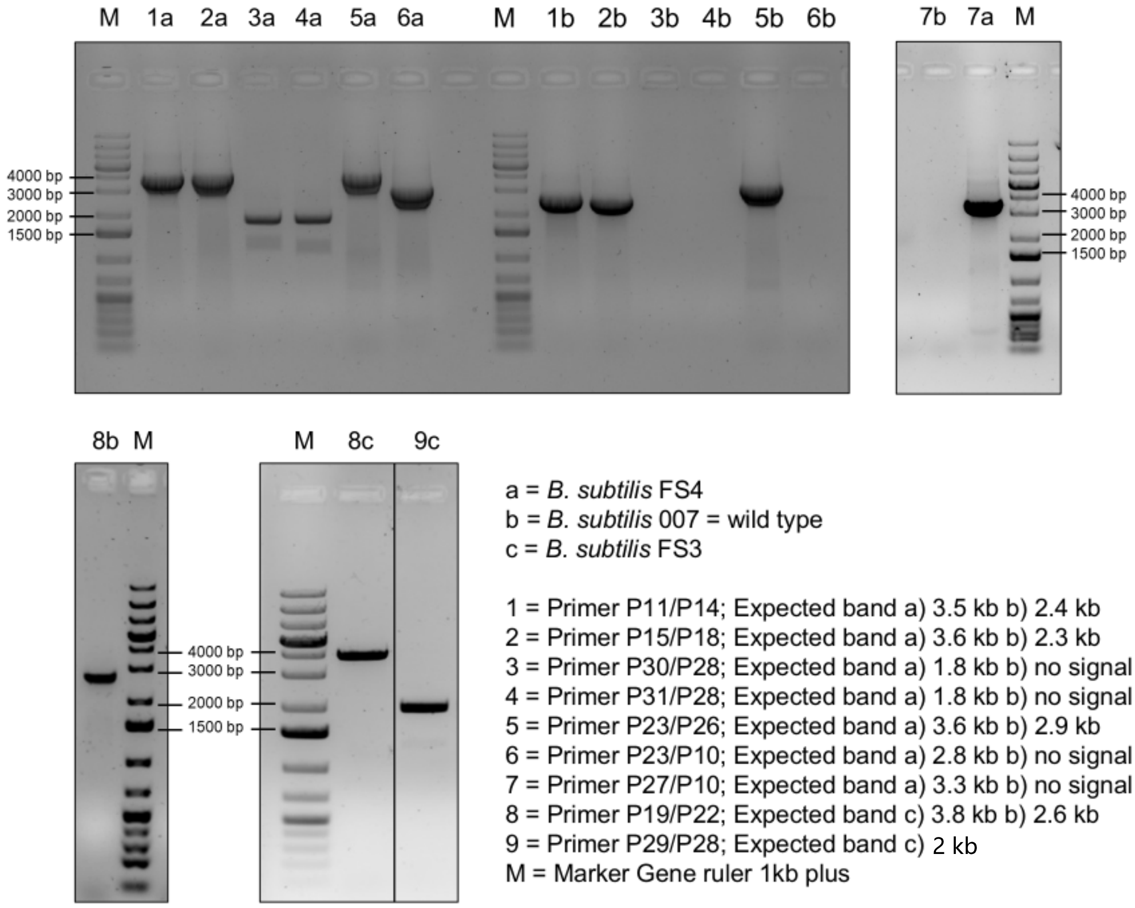

Supplemental Figure 1: Agarose gel of colony PCR for verification of *B. subtilis* FS4 and FS3. Primer pairs bind in upstream and downstream flank of *sigF* (1), *sfp* (2), *amyE* (5) and *flgE* (8); outside of the flanking region and inside of the PGB expression cassette of *sigF* (3), *sfp* (4), *amyE* (7) and *flgE* (9); in upstream flank and PGB expression cassette of *amyE* (6). The amplicons of 8c and 9c were used for sequencing.

A

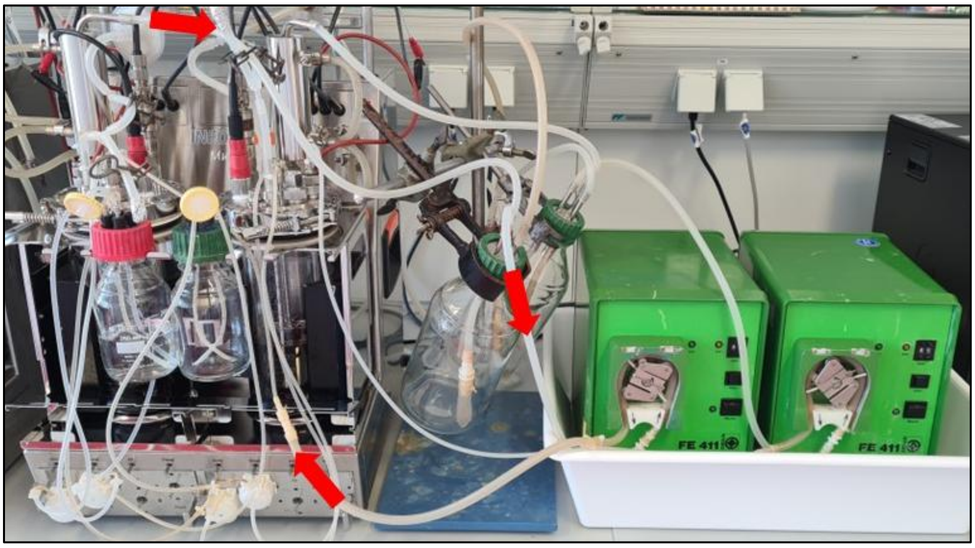

B

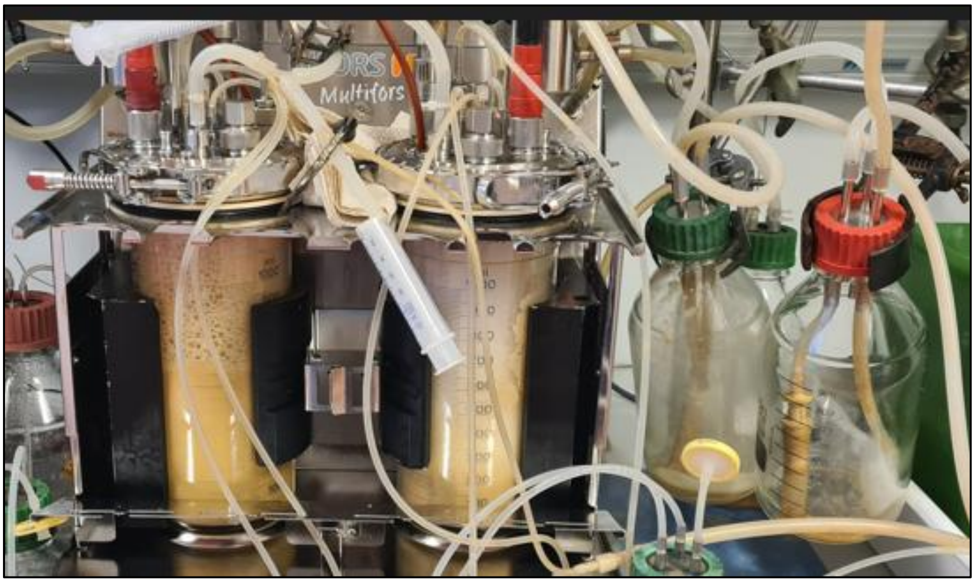

Supplemental Figure 2: Foam trap (A) and excessive foaming during bioreactor cultivation of *B. subtilis* FS1 (B). The red arrows indicate the direction of the foam pumped back into the reactors.

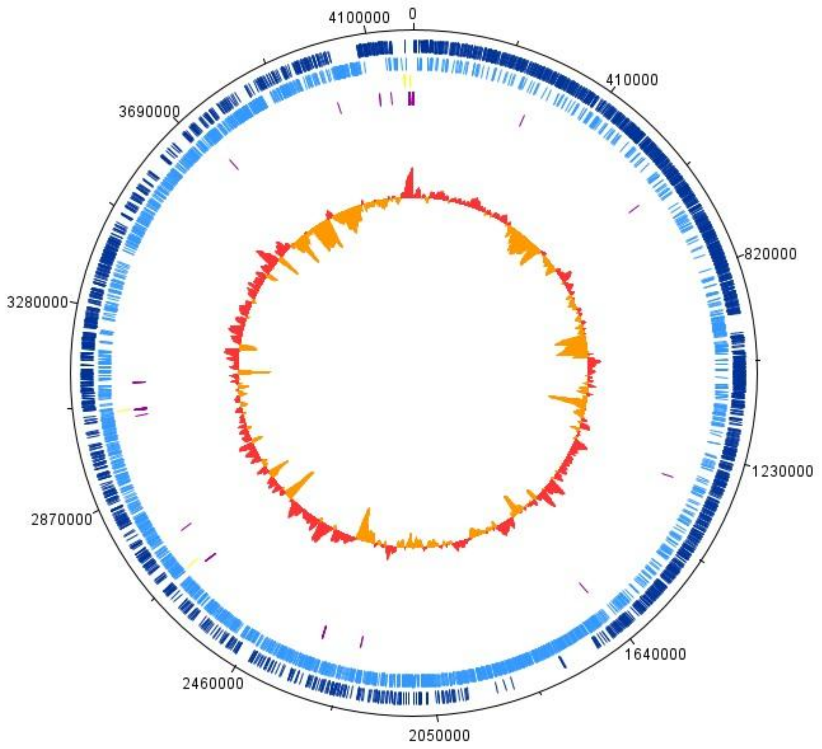

Supplemental Figure 3: Genome map of *B. subtilis* 007. CDS forward strand (dark blue); CDS reverse strand (blue); rRNA (yellow); tRNA (purple); GC plot below average (red) and above average (orange). The genome map was generated using DNA Plotter (Carver et al. 2009).

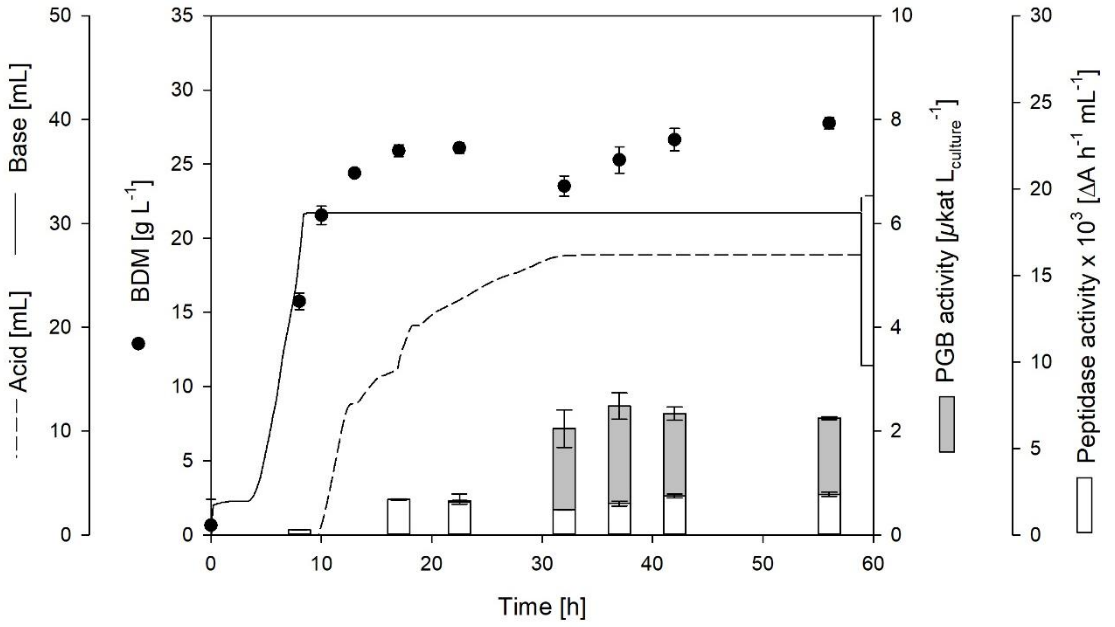

Supplemental Figure 4: Bioreactor cultivations of *B. subtilis* FS3.

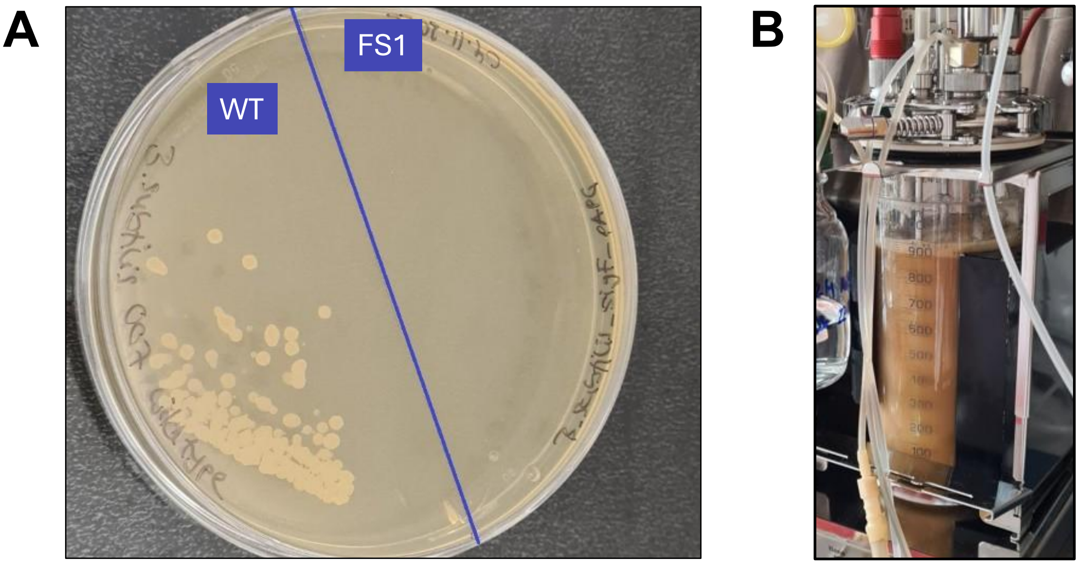

Supplemental Figure 5: Verification of the asporogenic phenotype of *B. subtilis* FS1 (A) and reduced foam formation in *B. subtilis* FS2 (B). (A) Overnight cultures of *B. subtilis* FS1 and the control strain *B. subtilis* 007 were heated for 10 min at 95°C and streaked on LB plates. Only sporulating cells survived the heat treatment and were able to grow, as shown for the control. **(B)** Excessive foaming was significantly reduced in *B. subtilis* FS2 during bioreactor cultivation.

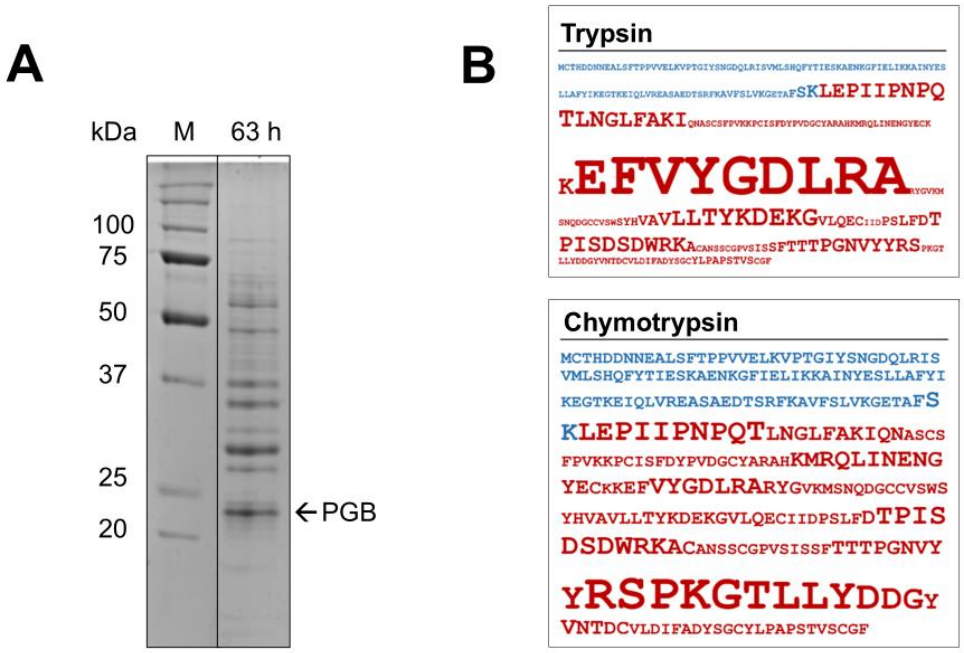

Supplemental Figure 6: SDS PAGE of culture supernatant from *B. subtilis* 007 carrying plasmid pLF_P_{aprE}_PhoD_pro-PGB (A) and MS analysis of the mature PGB band (B). (A) The culture supernatant after 63 h of shake flask cultivation was analyzed by SDS PAGE. 6 µg protein was loaded onto the gel. A dominant band at ~21 kDa was observed, corresponding to the size of the mature PGB (Eberhardt 2022). **(B)** The band between 20 and 25 kDa observed on SDS-PAGE during cultivations of *B. subtilis* 007 with plasmid pLF_P_{aprE}_PhoD_pro-PGB was analyzed by nano-liquid chromatography tandem mass spectrometry at the Core Facility Hohenheim (University of Hohenheim, Germany), as described previously (Senger et al. 2024, Horstmann et al. 2025). Mascot search results were imported into the Scaffold™ Software 4.10.0 (Proteome Software, USA). In-gel digestion was performed using trypsin or chymotrypsin. Fragment abundance is indicated by relative font size. The sequence of the mature PGB (red) was detected, whereas the pro-sequence (blue) was not.

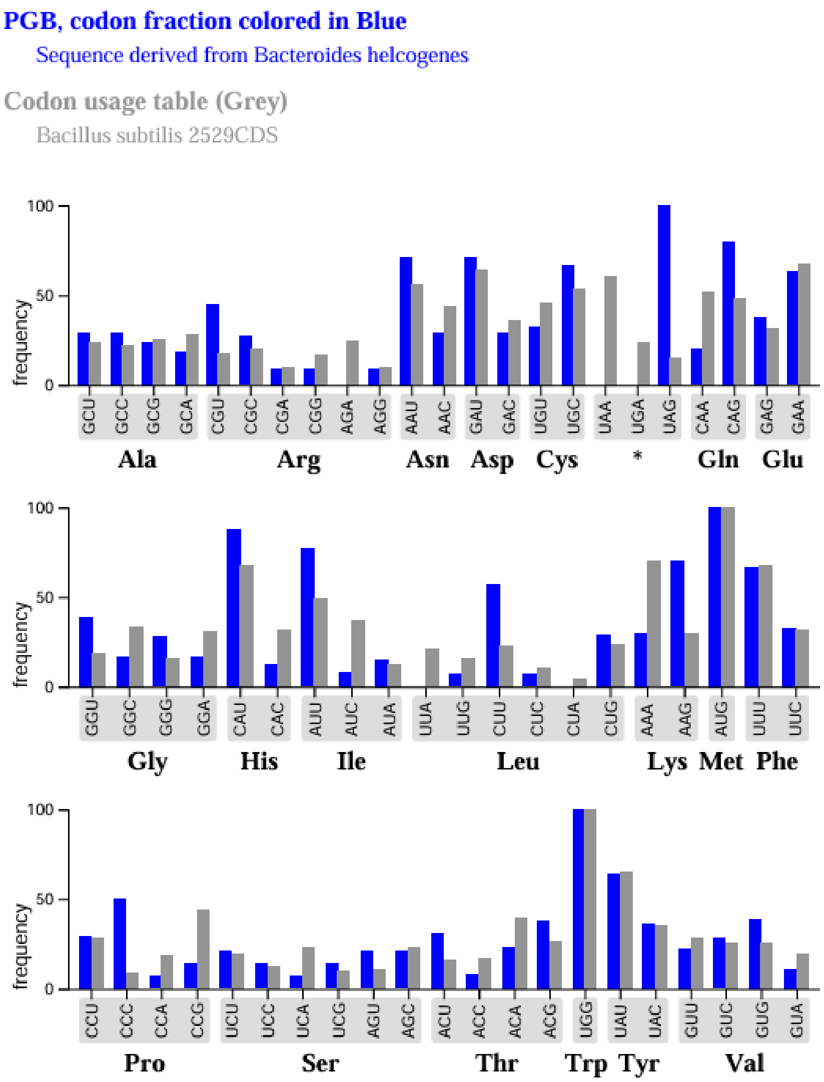

Supplemental Figure 7: Comparison of codon frequency in the native PGB gene with the codon usage in *B. subtilis*. The codons of the native PGB sequence (GenBank Accession: CP002352.1; Protein ID: ADV44662.1) was compared to the *B. subtilis* codon usage table using the GCUA (General Codon Usage Analysis) tool (McInerney 1998).

Sfp

```
CLUSTAL O(1.2.4) multiple sequence alignment


168_sfp/2       MKIYGIYMDRPLSQEENERFMSFISPEKREKCRRFYHKEDAHRTLLGDVLVRSVISRQYQ   60
168_sfp/1       ------------------------------------------------------------   0
007_sfp         MKIYGIYMDRPLSQEENERFMSFISPEKREKCRRFYHKEDAHRTLLGDVLVRSVISRQYQ   60
NRS6181_sfp     MKIYGIYMDRPLSQEENERFMSFISPEKREKCRRFYHKEDAHRTLLGDVLVRSVISRQYQ   60
MB8B10_sfp      MKIYGIYMDRPLSQEENERFMSFISPEKREKCRRFYHKEDAHRTLLGDVLVRSVISRQYQ   60


168_sfp/2       LDKSDIRFSTQEYGKPCIPDLPDAHFNISHSGRWVICAFDSQPIGIDIEKTKPISLEIAK   120
168_sfp/1       ------------------------------------------------------------   0
007_sfp         LDKSDIRFSTQEYGKPCIPDLPDAHFNISHSGRWVIGAFDSQPIGIDIEKTKPISLEIAK   120
NRS6181_sfp     LDKSDIRFSTQEYGKPCIPDLPDAHFNISHSGRWVIGAFDSQPIGIDIEKTKPISLEIAK   120
MB8B10_sfp      LDKSDIRFSTQEYGKPCIPDLPDAHFNISHSGRWVIGAFDSQPIGIDIEKTKPISLEIAK   120


168_sfp/2       RFFSKTEYSDLLAKDKDEQTDYFYHLWSMKESFIKQGRQRLIASA---------------   165
168_sfp/1       -------------------------------------------------MRLHQDGQVSI   11
007_sfp         RFFSKTEYSDLLAKDKDEQTDYFYHLWSMKESFIKQEGKGLSLPLDSFSVRLHQDGQVSI   180
NRS6181_sfp     RFFSKTEYSDLLAKDKDEQTDYFYHLWSMKESFIKQEGKGLSLPLDSFSVRLHQDGQVSI   180
MB8B10_sfp      RFFSKTEYSDLLAKDKDEQTDYFYHLWSMKESFIKQEGKGLSLPLDSFSVRLHQDGQVSI   180


168_sfp/2       --------------------------------------------   165
168_sfp/1       ELPDSHSPCYIKTYEVDPGYKMAVCAVHPDFPEDITMVSYEELL   55
007_sfp         ELPDSHSPCYIKTYEVDPGYKMAVCAAHPDFPEDITMVSYEELL   224
NRS6181_sfp     ELPDSHSPCYIKTYEVDPGYKMAVCAAHPDFPEDITMVSYEELL   224
MB8B10_sfp      ELPDSHSPCYIKTYEVDPGYKMAVCAAHPDFPEDITMVSYEELL   224
```

SwrA

```
CLUSTAL O(1.2.4) multiple sequence alignment


168_swrA2       MLVSTPVLLLILARAYLYKENGGSQLKRASIVREKKIL----------------------   38
168_swrA1       ------------------------------MCVKKKYYELVEQLKDRTQDVTFSATKALS   30
007_swrA        MLVSTPVLLLILARAYLYKENGGSQLKRASIVREKKYYELVEQLKDRTQDVTFSATKALS   60
NRS6181_swrA    MLVSTPVLLLILARAYLYKENGGSQLKRASIVREKKYYELVEQLKDRTQDVTFSATKALS   60
                                              :  :**

168_swrA2       ------------------------------------------------------------   38
168_swrA1       LLMLFSRYLVNYTNVESVNDINEECAKHYFNYLMKNHKRLGINLTDIKRSMHLISGLLDV   90
007_swrA        LLMLFSRYLVNYTNVESVNDINEECAKHYFNYLMKNHKRLGINLTDIKRSMHLISGLLDV   120
NRS6181_swrA    LLMLFSRYLVNYTNVESVNDINEECAKHYFNYLMKNHKRLGINLTDIKRSMHLISGLLDV   120


168_swrA2       ----------------------         38
168_swrA1       DVNHYLKDFSLSNVTLWMTQER         112
007_swrA        DVNHYLKDFSLSNVTLWMTQER         142
NRS6181_swrA    DVNHYLKDFSLSNVTLWMTQER         142
```

GudB

```
CLUSTAL O(1.2.4) multiple sequence alignment


168_GudB        MAADRNTGHTEEDKLDVLKSTQTVIHKALEKLGYPEEVYELLKEPMRLLTVKIPVRMDDG      60
007_GudB        MAADRNTGHTEEDKLDVLKSTQTVIHKALEKLGYPEEVYELLKEPMRLLTVKIPVRMDDG      60
                ************************************************************

168_GudB        SVKIFTGYRAQHNDSVGPTKGGIRFHPNVTEKEVKAVKALSIWMSLKCGIIDLPYGGGKG      120
007_GudB        SVKIFTGYRAQHNDSVGPTKGGIRFHPNVTEKEVKA---LSIWMSLKCGIIDLPYGGGKG      117
                ************************************   *********************

168_GudB        GIVCDPRDMSFRELERLSRGYVRAISQIVGPTKDVPAPDVFTNSQIMAWMMDEYSRIDEF      180
007_GudB        GIVCDPRDMSFRELERLSRGYVRAISQIVGPTKDVPAPDVFTNSQIMAWMMDEYSRIDEF      177
                ************************************************************

168_GudB        NSPGFITGKPLVLGGSHGRESATAKGVTICIKEAAKKRGIDIKGARVVVQGFGNAGSYLA      240
007_GudB        NSPGFITGKPLVLGGSHGRESATAKGVTICIKEAAKKRGIDIKGARVVVQGFGNAGSYLA      237
                ************************************************************

168_GudB        KFMHDAGAKVVGISDAYGGLYDPEGLDIDYLLDRRDSFGTVTKLFNDTITNQELLELDCD      300
007_GudB        KFMHDAGAKVVGISDAYGGLYDPEGLDIDYLLDRRDSFGTVTKLFNDTITNQELLELDCD      297
                ************************************************************

168_GudB        ILVPAAIENQITEENAHNIRAKIVVEAANGPTTLEGTKILSDRDILLVPDVLASAGGVTV      360
007_GudB        ILVPAAIENQITEENAHNIRAKIVVEAANGPTTLEGTKILSDRDILLVPDVLASAGGVTV      357
                ************************************************************

168_GudB        SYFEWVQNNQGFYWSEEEVEEKLEKMMVKSFNNIYEMANNRRIDMRLAAYMVGVRKMAEA      420
007_GudB        SYFEWVQNNQGFYWSEEEVEEKLEKMMVKSFNNIYEMANNRRIDMRLAAYMVGVRKMAEA      417
                ************************************************************

168_GudB        SRFRGWI   427
007_GudB        SRFRGWI   424
                *******
```

Supplemental Figure 8: Sequence alignment of SwrA, Sfp and GudB of *B. subtilis* 007 and *B. subtilis* 168, NRS6181 or MB8_B10.

References

Carver T, Thomson N, Bleasby A, Berriman M, Parkhill J (2009) DNAPlotter: circular and linear interactive genome visualization. Bioinformatics 25:119-20. https://doi.org/10.1093/bioinformatics/btn578

McInerney JO (1998) GCUA: general codon usage analysis. Bioinformatics 14:372-3. https://doi.org/10.1093/bioinformatics/14.4.372.

Senger J, Seitl I, Pross E, Fischer L (2024) Secretion of the cytoplasmic and high molecular weight *β*-galactosidase of *Paenibacillus wynnii* with *Bacillus subtilis*. Microb Cell Fact 23:70. https://doi.org/10.1186/s12934-024-02445-7.

Horstmann G, Roth N, Pross E, Seitl I, Fischer L (2025) In-vitro activation of recombinant pro-protein-glutamine glutaminases from *Bacteroides helcogenes* and *Flavobacterium* sp. Mol Catal 572:114718. https://doi.org/10.1016/j.mcat.2024.114718

Eberhardt D (2022) Optimierung der sekretorischen Herstellung der Proteinglutaminase aus *Bacteroides helcogenes* mit *Bacillus subtilis*. Bachelor Thesis, University of Hohenheim, Stuttgart.
